# How Rare Are Argonautoidea Octopuses in the Mediterranean? New Data from Stranding Events, Stomach Contents and Genetics

**DOI:** 10.3390/biology12030420

**Published:** 2023-03-09

**Authors:** Pietro Battaglia, Cristina Pedà, Carmen Rizzo, Maria Giulia Stipa, Erika Arcadi, Francesco Longo, Giovanni Ammendolia, Mauro Cavallaro, Ignazio Rao, Alberto Villari, Rosario Calogero, Pierpaolo Consoli, Mauro Sinopoli, Franco Andaloro, Teresa Romeo

**Affiliations:** 1 Department of Integrative Marine Ecology (EMI), Stazione Zoologica Anton Dohrn, National Institute of Biology, Ecology and Marine Biotechnology, Sicily Marine Centre, c/o Villa Pace, Contrada Porticatello 29, 98167 Messina, Italy; 2 Department Ecosustainable Marine Biotechnology, Stazione Zoologica Anton Dohrn, National Institute of Biology, Ecology and Marine Biotechnology, Sicily Marine Centre, c/o Villa Pace, Contrada Porticatello 29, 98167 Messina, Italy; 3 Institute of Polar Sciences, National Research Council (CNR.ISP), Spianata S. Raineri 86, 98122 Messina, Italy; 4 Independent Researcher, Messina, Italy; 5 Museo della Fauna, Department of Veterinary Sciences, University of Messina, Polo Universitario dell’Annunziata, 98168 Messina, Italy; 6 Department of Integrative Marine Ecology (EMI), Stazione Zoologica Anton Dohrn, National Institute of Biology, Ecology and Marine Biotechnology, Sicily Marine Centre, Lungomare Cristoforo Colombo N. 4521 (ex Complesso Roosevelt) Località Addaura, 90149 Palermo, Italy; 7 Department of Integrative Marine Ecology (EMI), Stazione Zoologica Anton Dohrn, National Institute of Biology, Ecology and Marine Biotechnology, Sicily Marine Centre, Via dei Mille 46, 98057 Milazzo (ME), Italy; 8 ISPRA, Italian National Institute for Environmental Protection and Research, Via dei Mille 46, 98057 Milazzo (ME), Italy

**Keywords:** *Argonauta*, *Ocythoe*, *Tremoctopus*, occurrence, rarity, pelagic environment, predators, genetics

## Abstract

**Simple Summary:**

This study reports the results of a multidisciplinary research on Mediterranean pelagic octopods belonging to the species *Argonauta argo, Ocythoe tuberculata, Tremoctopus gracilis* and *Tremoctopus violaceus* for the first time. The study area was the Strait of Messina and southern Tyrrhenian Sea. We used information from stranding events, accidental fishing catches and stomach contents of large predators (albacore, bluefin tuna, swordfish and Mediterranean spearfish). We analysed 47 fresh octopods, including exceptional records of rare males, and 330 individuals found in the stomachs of 800 predators. The analysis of genetic aspects was used to provide further details on the life and identity of these species.

**Abstract:**

The present paper represents the first all-encompassing study on all Mediterranean holopelagic octopods belonging to Argonautoidea (*Argonauta argo, Ocythoe tuberculata, Tremoctopus gracilis, Tremoctopus violaceus*). Argonautoidea octopuses were collected by different sampling methods in the Strait of Messina and southern Tyrrhenian Sea. The aim of this paper was to improve knowledge, using information from different data sources, such as the study of stranded individuals or accidental caught specimens, as well as the analysis of stomach content of large pelagic fishes. Moreover, we investigated their taxonomic profile through the amplification of the mitochondrial cytochrome c oxidase subunit I (COI). Overall, 47 fresh holopelagic octopods were collected, including valuable records of rare males. Moreover, 330 Argonautoidea octopuses were found in the stomachs of 800 predators. The results provided evidence that these cephalopods are more abundant than thought in the past. The molecular approach supported the ecological results with interesting insights. The similarity-based identifications and tree-based methods indicated that three females could be identified as *Tremoctopus violaceus* in agreement with their morphological classifications. The sequences obtained from the two *T. gracilis* individuals were clustered with the sequences of *Tremoctopus violaceus* from the Gulf of Mexico and were differentiated from the sequences attributed to *T. gracilis* and *T. robsoni*. The study represents a valuable contribution to the genetic characterization of Mediterranean individuals of the genera *Tremoctopus*, *Argonauta* and *Ocythoe*.

## 1. Introduction

Argonautoidea are holopelagic octopuses distributed worldwide and characterized by a peculiar biology and ecology. This superfamily includes four monogeneric families (Alloposidae, Argonautidae, Ocythoidae, Tremoctopodidae) that contain the following species, respectively [1]: *Haliphron atlanticus* Steenstrup, 1861, *Argonauta argo* Linnaeus 1758, *Argonauta hians* Lightfoot 1786, *Argonauta nodosus* Lightfoot 1786, *Argonauta nouryi* Lorois 1852, *Ocythoe tuberculata* Rafinesque 1814, *Tremoctopus gelatus* Thomas, 1977, *Tremoctopus gracilis* (Souleyet, 1852), *Tremoctopus robsoni* Kirk, 1884, *Tremoctopus violaceus* delle Chiaje, 1830. 

In the Mediterranean Sea, only four Argonautoidea cephalopods have been reported, i.e., *A. argo, O. tuberculata* and *T. violaceus*, which are considered native Mediterranean species, and in addition the non-indigenous *T. gracilis*. The occurrence of *T. gracilis* was documented for the first time by Belluscio et al. [2] and Orsi Relini et al. [3] in the Tyrrhenian Sea, although, later, a careful analysis of the literature confirmed that this species had already been observed many years earlier by Kramer [4] in the Adriatic Sea, but was erroneously identified as *T. violaceus* [5,6]. The presence of *H. atlanticus* was instead excluded because it was not supported by scientific data [1,7,8,9].

All these species have been unfrequently observed and, for this reason, several aspects of their biology and ecology have been poorly investigated and remain almost unknown. This also implies that population structure and the abundance of these species in the Mediterranean basin cannot be narrowly assessed yet. Although holopelagic octopuses are often referred to as rare cephalopods, the definition and the concept of rarity should take into account the difficulty in sampling. Open pelagic waters are usually less investigated than coastal areas or benthic environments. Moreover, several uncommon species show an elusive behaviour towards conventional sampling methods, avoiding gears [10,11,12].

Furthermore, Argonautidae, Ocythoidae and Tremoctopodidae have a peculiar reproductive biology and behaviour and are characterized by an evident sexual dimorphism with large females and dwarf males. The estimation of their abundance and sex ratio is still a highly controversial question, the catch of males being a very rare event, although females’ mantle cavity or, in *Argonauta* oothecae, one or more male hectocotyli are often found [13,14]. Consequently, current scientific knowledge mainly concerns female individuals, whereas information on males is scarce. It has also been hypothesized that males may also be eaten by the females after releasing their hectocotylus, thus providing a surplus of energy to achieve proper egg maturation [14].

The difficulty in tracking the vertical and horizontal movements, distribution and abundance of elusive cephalopod species has prompted some researchers to use the stomach contents of large pelagic predators [15,16,17,18,19] or seabirds [20,21] to obtain useful data on several pelagic cephalopods. This method improves knowledge on these species, also providing information on the frequency distribution of cephalopod size, using the measures of lower beaks to reconstruct mantle length or biomass [18].

The aim of this paper is to improve the knowledge on Mediterranean Argonautoidea, using information from different data sources, such as the study of stranded individuals or accidental caught specimens, as well as the analysis of the stomach contents of large pelagic fishes. Moreover, a barcoding approach was used to obtain sequences of the mitochondrial gene for cytochrome c oxidase subunit I (COI), in order to support the species identification and to clarify some aspects of the taxonomy of these species.

## 2. Materials and Methods

### 2.1. Sampling

**Figure 1:**
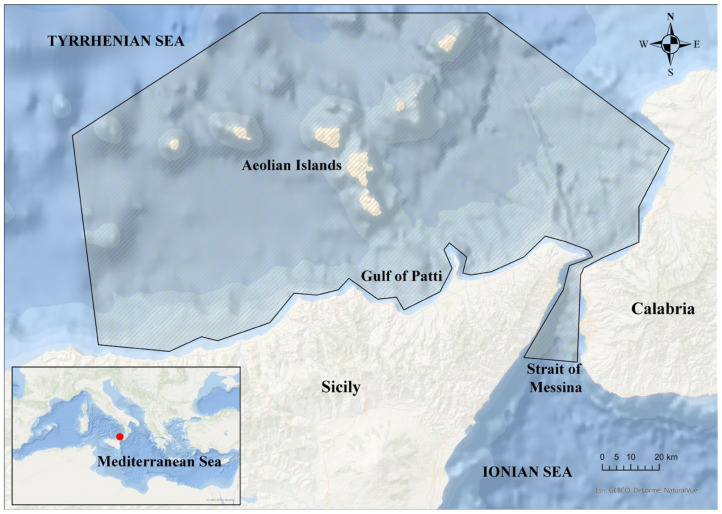
Study area including the southern Tyrrhenian Sea and the Strait of Messina. The delimited grey area indicates the study area.

Information on Argonautoidea octopuses were obtained by different sampling methods in the Strait of Messina and southern Tyrrhenian Sea (Figure 1). 

#### 2.1.1. Stranded Individuals

All fresh specimens of *A. argo* and also empty argonautid oothecae were found stranded on the Sicilian coast of the Strait of Messina, thanks to the particular hydrodynamic regime and upwelling currents of this area, which allows the stranding of mesopelagic, uncommon and rare fauna [22]. In some cases, waves washed the females onto the beach without their ootheca. Several other records are represented by the occurrence of empty oothecae. 

This biological material was collected before sunrise, in order to avoid dehydration and also predation by seabirds, ants and wasps [22]. From the first period of sampling, the scientific aim of our research was the monitoring of stranded mesopelagic fish; the date of the occurrence of holopelagic octopods was not always recorded. In addition, several individuals of *A. argo* were found still alive (approximately 30 specimens), and thus were returned to the sea.

Other stranded individuals belonged to *O. tuberculata* (*n* = 5) and *T. violaceus* (*n* = 10); furthermore, two males of *Tremoctopus* sp. were also found, whereas no specimens of *T. gracilis* were collected using this methodology. The list of stranded samples is reported in Table S1 of Supplementary Materials.

#### 2.1.2. Occasional Catches

A few individuals were also caught by recreational or professional fishermen using nets or hooks and lines, and were then delivered to researchers. In particular, three individuals of *O. tuberculata* were caught during recreational fishing trips targeting the European flying squid *Todarodes sagittatus* in the southern Tyrrhenian Sea, whereas a recent catch of *O. tuberculata* was obtained by trammel net in the same macroarea (details of this last catch was already described by Battaglia and Stipa [23]). The larger individuals of *T. gracilis* (*n* = 2) examined in this study were caught using hand nets by some recreational fishermen, in the southern Tyrrhenian Sea and the Strait of Messina, respectively. The most recent record dates back to 12 February 2022, when another specimen was caught with a combined trammel net-gillnet by professional fishermen in the Gulf of Patti (southern Tyrrhenian). From a total of 13 *T. violaceus*, only 3 specimens were collected by hand nets in the Strait of Messina. 

The list of samples caught by fishermen is reported in Table S1, together with the stranded individuals.

#### 2.1.3. Argonautoidea in Stomach Contents of Top Predators

Other samples were obtained by the analysis of the stomach contents of four large pelagic predators: albacore (*Thunnus alalunga*), the bluefin tuna (*Thunnus thynnus*), the Mediterranean spearfish (*Tetrapturus belone*) and swordfish (*Xiphias gladius*). The stomachs of these fish were collected at landings of commercial fishing (drifting long-lines or harpoons) between 1998 and 2021, during different research activities. The size of these predators was recorded by measuring fork length (FL) in albacore and bluefin tuna, and lower jaw fork length (LJFL) in Mediterranean spearfish and swordfish, respectively. Details of sampling periods, the number of specimens and size ranges of predators are given in Supplementary Materials (Table S2).

### 2.2. Morphometric and Biological Analysis 

Holopelagic octopuses were identified following taxonomical features available in the bibliography [6,13,24,25,26] and photographed. Dorsal mantle length (DML) was measured to the nearest 0.1 mm and, where possible, the body mass (W) was recorded to the nearest 0.01 g (some specimens collected several years ago had been already preserved in formalin or alcohol; a few stranded specimens were found damaged). Beaks were removed, cleaned and stored in a solution of ethanol, glycerine and water. The hood length of lower beaks (LHL) was measured to the nearest 0.1 mm. 

The relationship between LHL and DML was separately estimated through a linear regression equation for *A. argo, O. tuberculata* and *T. violaceus,* whereas it was not possible to calculate an equation for *T. gracilis* due to the low sample number. This analysis was only performed on female individuals, since we collected few males, and given the evident sexual dimorphism which characterizes these cephalopods. In order to improve the reliability of these equations, some additional data from the available literature [27,28,29,30,31,32,33] were retrieved and used for this purpose. 

*A. argo* ootheca is not a true molluscan shell. According to Power [34] and Finn [35], unlike the shells of other molluscs (e.g., gastropods), argonaut oothecae are not produced by the derivatives of the shell field, but it is a secondary calcium carbonate structure secreted from webs on the distal ends of the female argonaut’s first (dorsal) arm pair. In this paper, we use the term “ootheca” to indicate the brood case and the term “oothecae” for the plural. However, with the aim of providing comparable results, only for the morphometric features of the ootheca, we followed the terminology of Finn [13,25,35], which uses the word “shell” to indicate this structure in measurements.

*A. argo* oothecae (including both those belonging to stranded individuals and stranded empty oothecae) were measured to the nearest 0.1 mm by a caliper, recording the “shell” length (ShL), aperture length (ApL), “shell” breadth (ShB), aperture width (ApW) and ear width (EW) [25,35]. The relationship between the ShL and the other dimensions was estimated according to linear regression. Furthermore, linear regression was used to correlate the DML and ShL in *A. argo*.

### 2.3. Occurrence of Holopelagic Octopuses in Predator Diet 

The stomachs of predators were dissected and their contents were examined by visual sorting using a dissecting microscope. Holopelagic octopod prey items were identified according to the taxonomic features reported by [13,26]. Frequently, beaks were the only identifiable hard structures due to an advanced digestion process; in this case, the lower beaks were observed to identify the holopelagic octopuses at species level, according to the identification keys [27,29,36] and comparing them with our reference collection [37]. Each lower beak was measured to the nearest 0.1 mm, recording the lower hood length (LHL). 

The frequency of occurrence (%F) for each Argonautoidea species in the stomach of predators was calculated as follows:

%*Fi* = number of stomachs containing prey *i*/total number of examined stomachs × 100.

### 2.4. Genetic Analysis 

Genetic analysis was only performed on a part of samples, due to the conservation status of long-stored specimens. These analyses were devoted to the taxonomic identification of individuals, in support of morphological identification and as a contribution to the current existing knowledge about intra- and interspecific variability within the taxa.

#### 2.4.1. DNA Extraction and Amplification of Mitochondrial Cytochrome c Oxidase Subunit I (COI)

Total genomic DNA was extracted from the muscle of *Argonauta argo*, *Tremoctopus gracilis, Tremoctopus violaceus* and *Tremoctopus* sp., and/or hectocotyli of *Argonauta argo* and *Ocythoe tuberculata* specimens (see Table S3), using EZNA Tissue DNA Kit (Omega Bio-Tek) according to manufactory instructions. The DNA amount and quality were determined using a NanoDrop™ One (Thermo scientific, Thermo Fisher Scientific Inc., Dublin, UK) UV spectrophotometer. Two pairs of primers, Fish F2/Fish R2 (Fish F2, CAACCAACCACAAAGACATTGGCAC; Fish R2, ACTTCAGGGTGACCGAAGAATCGAA; [38] and dgLCO/dgHCO, were used to amplify the fragment of COI sequences of each specimen. PCR reactions were performed using a BioRad C100 touch thermocycler (BioRad Laboaratories) in a 25 μL reaction mixture final volume containing 12.5 μL 2x MyTaq HS (Bioline), 0.5 μL of each primer, 10.5 μL of sterile distilled water and 1 μL of diluted template DNA. The following cycling parameters were used: an initial step for 120 s at 94 °C, followed by 35 cycles at 94 °C for 30 s, at 50 °C for 60 s and at 72 °C for 95 s. The final extension step was at 72 °C for 420 s. The PCR products were visualized on 1% agarose gel electrophoresis.

#### 2.4.2. Sequencing and Molecular Analysis

Amplified products were sequenced by Eurofins genomic (Milan, Italy). The total COI region sequences were submitted to GenBank using Basic Local Alignment Search Tool (BLAST) [39] and were then aligned with highly similar sequences using the Clustal W algorithm implemented in MEGA v7 (http://multalin.toulouse.inra.fr/multalin/, accessed on 29 January 2021) [40]. COI sequences were also compared to available barcode records in BOLD, using the identification engine BOLD-IDS, with the option “COI Full Database”, as described by Agus et al. [41]. Sequences derived from a complete mitochondrial genome (AB158363), attributed to the species *Octopus vulgaris*, were used as an outgroup for both COI. Genetic divergences between the sequence obtained in this study and the closest relatives detected according to the Blast search from GenBank were calculated in MEGA 7 [42] using the Kimura two-parameter model (K2P) [43]. The homologous sequences from GenBank used in this study are listed in Table S4.

## 3. Results

### 3.1. Stranded Individuals and Occasional Catches

Overall, during the entire sampling period, 47 fresh specimens belonging to Argonautoidea were collected (Table S1). Most of them were found/caught in the Strait of Messina (87.2% of samples), and 42.6% were *A. argo*. Most individuals were collected during the spring and winter seasons (42.5% and 31.9%, respectively). With regard to stranded specimens, the percentage of findings during spring increased to 47.2% (Figure S1).

In the following sub-paragraphs, we report results for each investigated species.

#### 3.1.1. *Argonauta argo*

**Figure 2:**
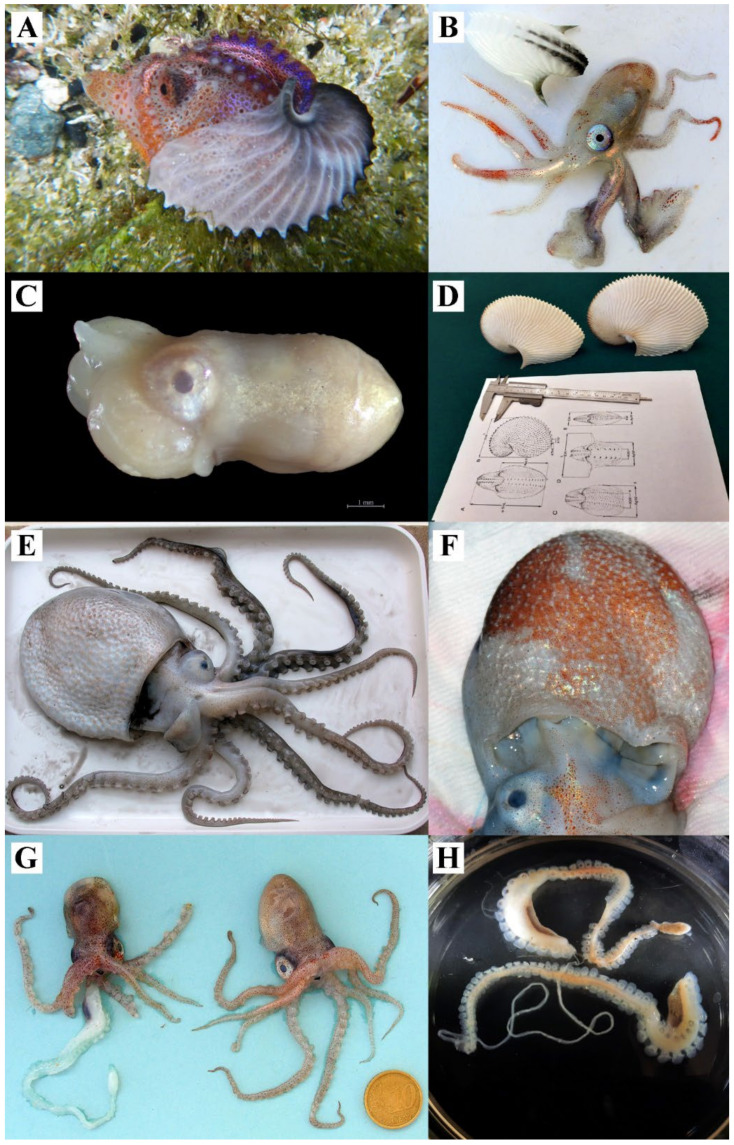
(**A**) Lateral view of a live female of *Argonauta argo* photographed in the Strait of Messina; (**B**) Female specimen of *A. argo* stranded in the Strait of Messina, this figure shows the first pair of dorsal arms used to secrete the ootheca; (**C**) A mature male of *A. argo* showing its hectocotylized arm enwrapped in a sac under the left eye; (**D**) Data collection (measurements) from *A. argo* oothecae in the laboratory; (**E**) *Ocythoe tuberculata* female caught by a recreational fisherman in 2018 (Southern Tyrrhenian Sea); (**F**) Details of the ventral mantle of *O. tuberculata*, showing skin ridges and tubercles; (**G**) Two specimens of *O. tuberculata* found in 2008 in the Strait of Messina. Mature male with hectocotylus (left) and young female (right); (**H**) two hectocotyli found in the mantle cavity of a female *O. tuberculata*.

All 20 specimens of *A. argo* were found stranded on the Sicilian coast of the Strait of Messina. They were mainly female individuals (Figure 2A,B), measuring from 17.0 to 86.5 mm DML (mean ± SD = 34.7 ± 19.6 mm) and having beaks with LHL ranging from 1.8 to 5.6 mm (mean ± SD = 2.8 ± 1.0 mm). Nine females had already deposited eggs inside their ootheca and, in only six cases, we also found one or two hectocotyli stored inside the ootheca (a total of eight hectocotyli). Across the entire sampling period, only one stranded mature male was collected (Figure 2C). This specimen was 6.2 mm DML and had its hectocotylus inside the sac under the left eye. 

The relationship between LHL and DML, estimated through linear regression for 22 specimens of *A. argo* (including samples from Smale et al. [29]), was DML = 20.03 LHL − 19.783 (Figure S2A; R^2^ = 0.9398).

**Figure 3:**
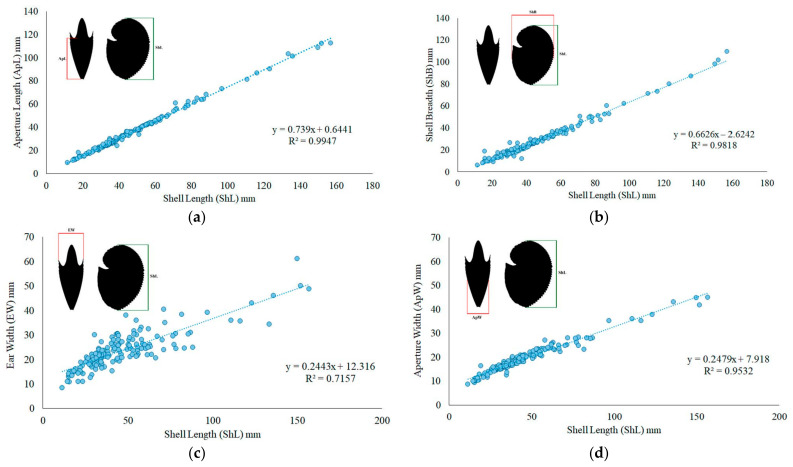
Morphometric relationships between the “shell” length (ShL) and: (**a**) aperture length (ApL); (**b**) “shell” breadth (Shb); (**c**) ear width (EW); (**d**) aperture width (ApW).

Overall, 164 paper nautilus oothecae were examined (Figure 2D), measuring from 11.6 to 157.0 mm ShL (mean ± SD = 46.8 ± 26.0 mm). The aperture length (ApL) varied between 9.4 and 112.3 mm (mean ± SD = 35.2 ± 19.2 mm), the “shell” breadth (ShB) between 5.9 and 109.4 mm (mean ± SD = 28.1 ± 16.9 mm) and the aperture width (ApW) between 8.7 and 45.0 mm (mean ± SD = 19.3 ± 6.4 mm), whereas the ear width (EW) ranged from 8.4 to 61.1 mm (mean ± SD = 23.7 ± 7.6 mm). The morphometric relationships of ShL (independent variable) against ApL, Shb, EW, ApW (dependent variable), respectively, are shown in Figure 3. The scatter plots indicated a high correlation between ShL against ApL, Shb and ApW; conversely, the relationship between ShL and EW showed a lower coefficient of determination (R^2^ = 0.7157). 

The relationship between the “shell” size (ShL) and female mantle length (DML) for 12 specimens of *A. argo* resulted as ShL = 1.435 DML − 4.042 (Figure S3; R^2^ = 0.946; additional data on four individuals from Guerra et al. [44]; Corsini et al. [45]; Kim et al. [46] and Giacalone et al. [47] were included in this analysis).

#### 3.1.2. *Ocythoe tuberculata*

Overall, nine specimens of *O. tuberculata* were collected in this study; five of them were found stranded on the coasts of the Strait of Messina, while four individuals were accidental catches recorded in the southern Tyrrhenian Sea. The eight females (Figure 2E,F) ranged from 25.0 to 318.0 mm DML (mean ± SD = 116.0 ± 94.3 mm); the largest specimen, caught by an artisanal fisherman using a trammel net, has already been described in a recent paper [23] and represents one of the largest individuals found in the Mediterranean basin. On the contrary, our samples include only a mature male individual, which stranded in the Strait of Messina together with a young female of the same species (Figure 2G). However, two large hectocotyli (Figure 2H) were also found in the mantle cavity of a mature female. They measured 118 and 109 mm (excluding the penile filament), and had 96 and 102 suckers, respectively. The first hectocotylus had a penile filament 214 mm long, while, in the second, the filament was wrapped inside its sac (Figure 2H). The relationship between LHL and DML, estimated through linear regression for 22 specimens of *O. tuberculata*, was DML = 19.127 LHL − 34.977 (Figure S2B; R^2^ = 0.935). This estimation included our samples and data from individuals collected by other studies [27,28,29,30,31,33,48,49].

#### 3.1.3. *Tremoctopus violaceus* and *Tremoctopus gracilis*

**Figure 4:**
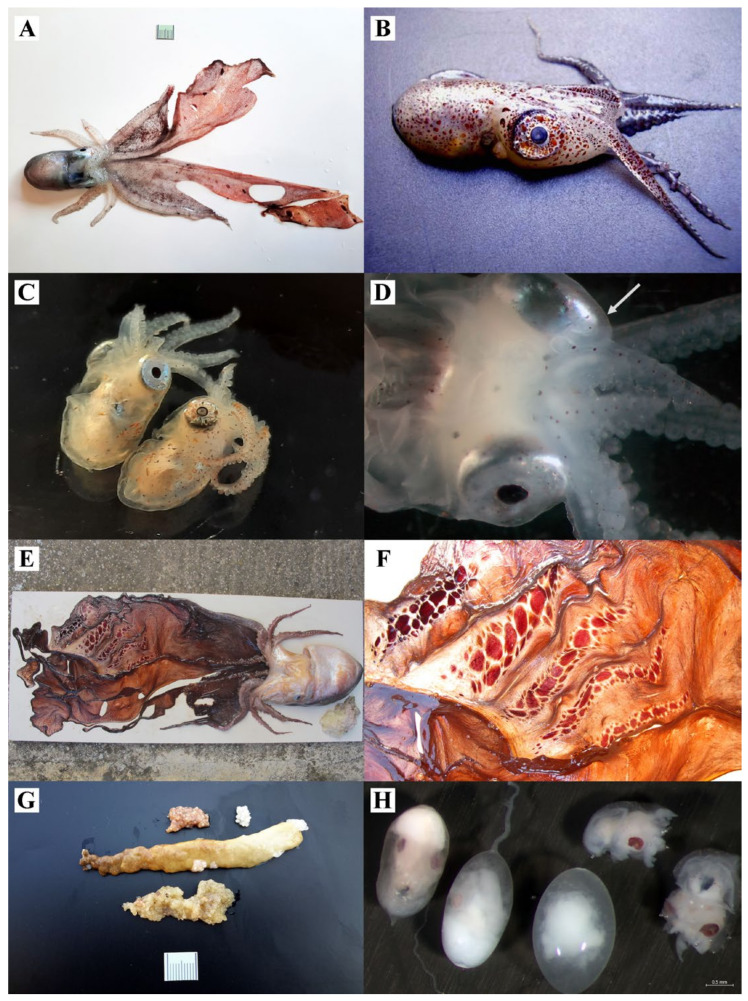
(**A**) Young female of *Tremoctopus violaceus*; (**B**) Mature male of *T. violaceus* with the hectocotylus inside in a sac under the right eye; (**C**) Two immature males of *Tremoctopus* spp. stranded in 2021 in the Strait of Messina; (**D**) Particular of the hectocotylus of an immature male of *Tremoctopus* spp. (the same individual photographed in Figure 4C, on the left) in a developing status within the sac under the right eye (ventral view, grey arrow). (**E**) Ventral view of a large mature female of *Tremoctopus gracilis*; (**F**) Web of dorsal arms of *T. gracilis* and chromatophore pattern typical of the species.; (**G**) Fertilized eggs of *T. gracilis* at different maturity stages and rod (centre) to which the egg mass is usually attached; (**H**) Fertilized eggs at different stages of development and embryos observed with a stereomicroscope.

All examined individuals of *T. violaceus* (*n* = 13) were found stranded in the Strait of Messina. The 12 females (Figure 4A) ranged from 7 to 161 mm DML (mean ± SD = 51.3 ± 52.0 mm), whereas the mantle length of the male individual (Figure 4B) was 26.1 mm. This male was mature and its hectocotylus was wrapped inside a voluminous sac under the right eye (Figure 4B). Two other males (Figure 4C) were immature and the development of their hectocotylus under the right eye was barely visible (Figure 4D). Therefore, it was not possible to discriminate which *Tremoctopus* species they belonged to.

The equation describing the relationship between beak and mantle size was DML = 21.93 LHL − 0.8597 (Figure S2C; R^2^ = 0.9352). We also included the two undetermined males in this analysis.

Overall, three female individuals of *T. gracilis* were collected. The largest individual (DML = 166 mm) was caught by hand nets in coastal waters of Milazzo (southern Tyrrhenian Sea), whereas the other large female (DML = 150 mm; Figure 4E,F) was sampled in the Strait of Messina using the same sampling gear. Recently, another small individual was caught by a professional fisherman using a combined trammel net–gillnet in the Gulf of Patti. In Figure 4E,F, the typical colour pattern of the web of dorsal arms can be observed. The specimen measuring 150 mm DML had a large egg mass, attached to the rod (Figure 4E,G) held within the arm crown, and eggs were characterized by different stages of maturity, also with embryos ready to hatch (Figure 4H). The eggs also displayed a different colour pattern depending on their stage of development (Figure 4G,H).

### 3.2. Occurrence of Holopelagic Octopuses in Predator Diets

A total of 800 stomachs of top predators were examined. Overall, 330 Argonautoidea octopuses were found in the stomachs of 123 specimens (%F = 15.4): 25 in albacore (%F = 9.4), 126 in bluefin tuna (%F = 16.9), 88 in Mediterranean spearfish (%F = 28.8) and 91 in swordfish (%F = 10.7).

Figure S4 summarizes the number of individuals for each Argonautoidea prey found in the stomach of large pelagic predators. *A. argo* was eaten by all predators, but it was more abundant in the guts of *T. belone* (*n* = 74) and *T. thynnus* (*n* = 59). *O. tuberculata* was not found in *T. belone* and was most abundant in the stomach contents of *X. gladius* (*n* = 26). The highest number of *Tremoctopus* spp. prey was eaten by *T. thynnus* (*n* = 61), whereas this food item was not ingested by *T. alalunga*. 

**Figure 5:**
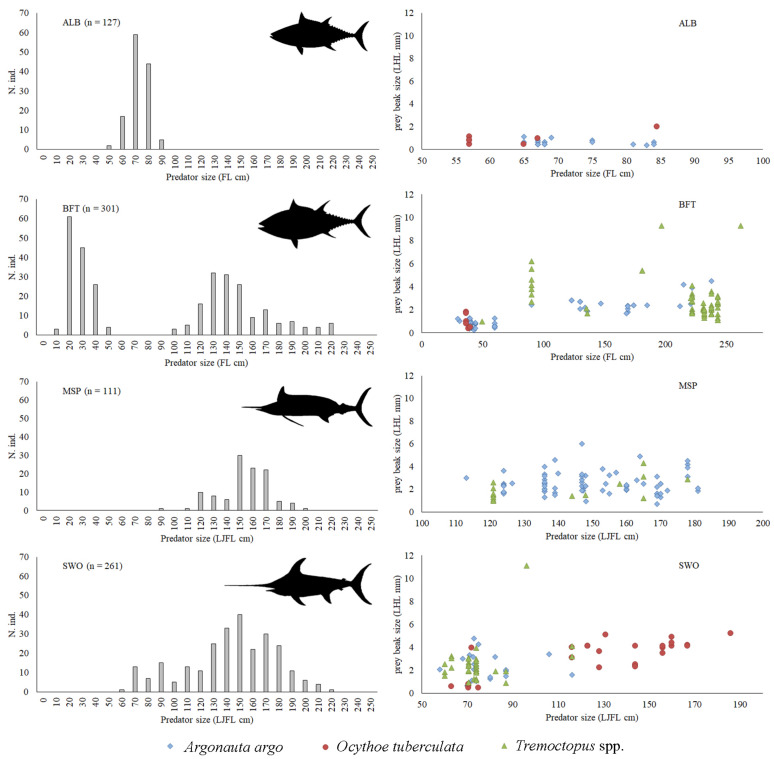
Histograms showing the size frequency distribution for individuals of pelagic predators (ALB = albacore; BFT = bluefin tuna; MSP= Mediterranean spearfish; SWO = swordfish), the stomachs of which were examined. Scatterplots showing the relationship between predator size (fork length FL or lower jaw fork length LJFL) and the lower beak size (LHL) of cephalopod food items belonging to Argonautoidea species.

Figure 5 shows both the size frequency distribution of each pelagic predator (ALB, BFT, MSP, SWO), the stomach contents of which were examined, and the relationship between predator size and prey lower beak size for each Argonautoidea species. No evidence of size-related predation on holopelagic octopuses was observed for these predators, except for *T. alalunga* (*n* = 127; FL range = 38.9–102.1 cm), which only preyed upon a few small individuals of *A. argo* (LHL from 0.38 to 1.11 mm) and *O. tuberculata* (LHL from 0.47 to 2 mm).

*Tremoctopus* spp. and *A. argo* were the most abundant holopelagic octopuses (61 *Tremoctopus* spp. against 59 *A. argo*) in stomachs of *T. thynnus* (*n* = 301; FL range = 6.3–222 cm). Specimens of *O. tuberculata* (LHL from 0.38 to 1.8 mm) were only eaten by small bluefin tuna, whereas adults mainly preyed upon *Tremoctopus* spp.

Despite the fact that we examined a lower number of stomachs of *T. belone* (*n* = 111; LJFL range = 105-196 cm), a relatively high amount of holopelagic octopus prey was observed. This predator showed a preference for *A. argo* (79 individuals overall ranging from 0.7 to 6 mm LHL), although *Tremoctopus* spp. was also ingested.

The analysis of the prey beak size *vs* predator length relationship indicated a dominance of *O. tuberculata* in larger individuals of *X. gladius*. This predator (*n* = 261; LJFL range= 72–216 cm) also preyed upon *A. argo* and *Tremoctopus* spp., but only swordfish ranging from 58 to 116 LJFL cm had these food items in their stomachs. 

### 3.3. Genetic Analysis

The COI sequences obtained using two different sets of primers were deposited in the GenBank database (Accession numbers: OP132805-OP132821). Some difficulties were encountered during COI gene amplification and sequencing, due to the low quality and quantity of the extracted DNA. This was the case of some egg and some hectocotyli samples, for which the COI amplification failed. All the results obtained with the two sets of primers are shown in Table 1 and Table 2. 

The BLAST and BOLD similarity searches generally agreed with the morphological identification obtained for all samples, with only some exceptions, as explained below. Molecular analysis performed with the primer set Fish F2/Fish R2 did not obtain COI sequences useful for the genetic identification of *Argonauta* spp. individuals. Conversely, the same primer set supported morphological identification of the sample OT005E, for which good matches with *O. tuberculata* were found in GenBank and BOLD databases (similarity 100%; GenBank accession number AY557519; BOLD accession number: KY947106).

Among *Tremoctopus* spp. individuals, the COI sequences obtained with the primer set Fish F2/Fish R2 for the samples TV011, TG002 and TV006 species showed higher similarities, with the sequence *T. violaceus* having the GenBank accession number of OM025233. The BOLD-IDS also showed correspondence with the species *T. violaceus,* but a higher similarity (97%) was detected with three private sequences. 

The primer set dgLCO/dgHCO obtained higher quality and quantity of the extracted DNA, even if, in this case, the taxonomical identification for DNA extracted from egg was not obtained; however, some hectocotyli samples were properly sequenced. The GenBank search for COI sequences obtained from the samples AA005, AA007, AA008 and AA015 showed higher similarity, with the sequence *A. argo* having the GenBank accession number LC596061; the COI sequence of sample AA006 presented a greater match with *A. argo* (AN:AB191273) and the DNA extracted from hectocotylus of the same sample showed the best similarity with the sequence *A. argo* (AN:LC596061). The BOLD-IDS search confirmed the taxonomic identification for samples A006 and A007 by matching them with two different deposited sequences, namely BOLD:AAJ825 and BOLD:AAJ8256, respectively. For the remaining *Argonauta* spp. individuals, the BOLD-ITS search showed higher matches with a private sequence of *A. hians* (similarities ranging between 98 and 100%). 

Similarity accounting for 99% was obtained from GenBank for samples OT005, with closest relative *O. tuberculata* (AN:KY947106), while the COI sequences obtained from the hectocotylus of *Ocythoe* sample OT005E showed 100% similarity with *O. tuberculata* (AN:AY557519), also confirmed by affiliation through BOLD-IDS search (BOLD:ADR2058).

The COI sequence obtained from samples TV006 and TV011 showed higher similarity with *T. violaceus* (AN: MW351787), while from sample TV008 the closest relative was *T. violaceus* (AN: OM025233). Finally, the COI sequences obtained from individuals of *T. gracilis* TG002 and TG003 showed the best similarity with next relatives *T. violaceus* MW351787. The similarity with the closest relatives affiliated to the species *T. gracilis* was 91% and 88% for samples TG002 and TG003, respectively (AN: MN370032). The BOLD-IDS search confirmed the same taxonomic identification for samples TV008 and TG002, but with a private sequence. BLAST pairwise alignments of *Tremoctopus* spp. COI sequences were performed to assess the similarity between sequences of our *Tremoctopus* samples. 

**Figure 6:**
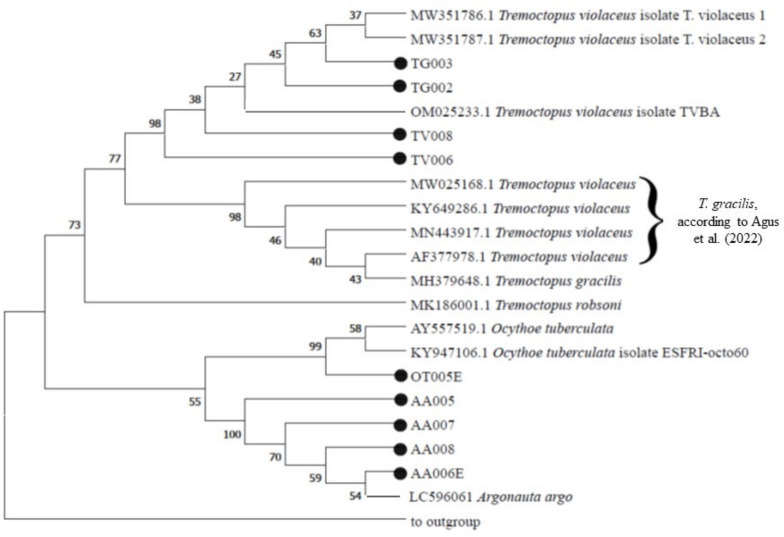
The phylogenetic tree was inferred using the Maximum Likelihood method and Kimura 2-parameter model [37]. The bootstrap consensus tree inferred from 1000 replicates is taken to represent the evolutionary history of the taxa analysed. The percentage of replicate trees in which the associated taxa clustered together in the bootstrap test (1000 replicates) are shown next to the branches. This analysis involved 22 nucleotide sequences. Sequences derived from a complete mitochondrial genome (NC_006353.1), attributed to the species *Octopus vulgaris*, was used as the outgroup, as reported in Agus et al. [41].

To assess the relationship between our query sequences and their neighboring reference sequences, the COI sequences obtained in this study were used to perform a phylogenetic analysis with the homologous sequences available in the public repositories. Considering all the *Tremoctopus*, *Argonauta* and *Ocythoe* sequences, the final alignments comprised 35 COI sequences, as showed in the phylogenetic tree (Figure 6). 

Two different phylogenetic trees were computed, one including a total of 22 nucleotide sequences, with our *Argonauta*, *Ocythoe* and *Tremocotpus* sequences and their closest relatives, and one involving fourteen nucleotide sequences for *Tremoctopus* sp. The shorter COI sequences obtained in this study were not used for phylogenetic trees, to avoid bias in the alignments (namely OT005, TV011, AA015, AA006).

**Figure 7:**
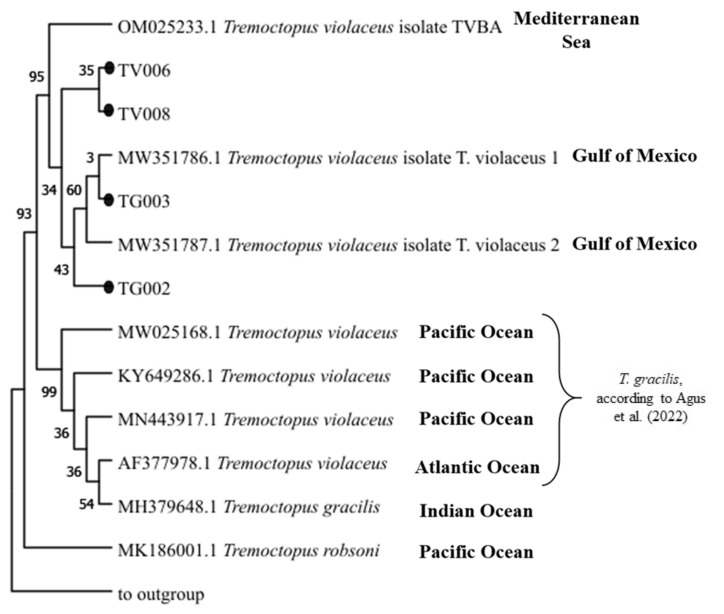
The phylogenetic tree was inferred using the Maximum Likelihood method and Kimura 2-parameter model [37]. The bootstrap consensus tree inferred from 1000 replicates is taken to represent the evolutionary history of the taxa analysed. The percentage of replicate trees in which the associated taxa clustered together in the bootstrap test (1000 replicates) are shown next to the branches. This analysis involved 14 nucleotide sequences. Sequences derived from a complete mitochondrial genome (NC_006353.1), attributed to the species *Octopus vulgaris*, was used as the outgroup, as reported in Agus et al. [41].

The phylogenetic tree in Figure 6 shows two main clades; one included two sub-clades with the sequences affiliated to *Argonauta* spp. and *Ocythoe* spp. The branch included our *A. argo* AA005, AA006E, AA007 and AA008, closely related to *A. argo* LC596061. The second branch included the individual OT005, closely related to next relatives *O. tuberculata* KY947106 and AY557519. An individual branch included all *Tremoctopus* spp. individuals. A separate phylogenetic tree was also computed only with sequences of *Tremoctopus* spp. individuals and their next relatives (Figure 7). 

As shown in Figure 7, the phylogenetic tree constituted three main branches: one included the reference sequence of *T. robsoni*; the second included one reference sequence deposited as *T. gracilis* and four reference sequences deposited in GenBank as *T. violaceus*, but reclassified by Agus et al. [41] as *T. gracilis*; a third branch included two sub-branches, one with the compiled sequences obtained in this study for TV006 and TV008, highly similar to the *T. violaceus* homologue sequence from the Mediterranean Sea (OM025233), and one with the sequences from our individuals *T. gracilis* TG002 and TG003 clustering with the two homologue sequences from the Gulf of Mexico (MW351786 and MW351787) (Table S4). To the best of our knowledge, these are among the first haplotypes of cytochrome c oxidase subunits I (COI) of *Tremoctopus* sp. from the Mediterranean Sea publicly available in GenBank, following the study of Agus et al. [41].

## 4. Discussion

The present paper represents the first all-encompassing study on all Mediterranean holopelagic octopods belonging to Argonautoidea. The results improve the knowledge on these species, through a multidisciplinary approach, addressing morphological, ecological and genetic aspects. 

Despite the stranding of a few specimens of *A. argo*, *O. tuberculata* and *T. violaceus* reported in previous years [50,51,52], our monitoring activity of the phenomenon of fauna stranding in the Strait of Messina allowed us to collect a good number of samples, including valuable records of rare males. Overall, one male of *A. argo*, one male of *O. tuberculata,* one male of *T. violaceus* and two males of *Tremoctopus* spp. were examined. Unfortunately, these last two specimens were immature and the hectocotylus had scarcely developed; it was not possible to discriminate whether they were *T. violaceus* or *T. gracilis*; their identification being based on the counts of the proximal and distal suckers of the hectocotylus. However, the genetic analysis suggests that these two males may be attributed to the species *T. violaceus*. Curiously, the male of *O. tuberculata* was found stranded in proximity to a small and immature female of the same species, but it was not certain they were close when they were at sea. The finding of males belonging to all known Mediterranean species is an exceptional event; indeed, up to date and according to our knowledge, observations of Argonautoidea males in Mediterranean waters have been rarely reported. In the past, a few authors have published some drawings showing males of these species (e.g., [53,54,55]); only Bello [56] published the photo of a male of *T. violaceus* found stranded in the Strait of Messina by Alberto Villari (one of the co-authors of this paper), examined here. Other observations were limited to the finding of male hectocotyli in a female’s mantle or argonaut ootheca (e.g., [12,14,54,57]). In a previous paper [14], the finding of a vital hectocotylus inside an *A. argo* ootheca was discussed, reporting interesting observations on its morphology (the penile filament was contained in a special sac and the hectocotylus assumed a folded position to protect it) and behaviour, underlining the ootheca function in storing the hectocotyli received by males until copulation. Moreover, the high vitality and endurance of the hectocotylus after detachment from the male represented an added value for reproductive success [14]. During the monitoring activity of the present study, some hectocotyli of *A. argo* (exclusively found inside the oothecae) and *O. tuberculata* (found in the mantle cavity) were collected, whereas none were found in *Tremoctopus* spp. The finding of *O. tuberculata*’s hectocotyli measuring 118 and 109 mm suggests that males of this species may reach larger sizes than previously assumed or observed so far; indeed, Jereb et al. [13] reported a maximum length of 30 mm mantle length and 69 mm total length for males. Regarding females, the specimens examined here included a large *O. tuberculata,* previously reported by Battaglia and Stipa (in Tsagarakis et al. [58]), representing the second largest specimen ever (318 mm ML and 4835 g of total weight), after the individual recorded by Salman and Akalin [33] in the Aegean Sea (335 mm ML and 5060 g of total weight). 

The seasonality of Argonautoidea occurrence in the study area suggested that, in general, the probability of encountering these cephalopods is higher during spring and winter, although some differences were observed between species. For instance, most stranded individuals of *A. argo* were found in spring and autumn, whereas *T. violaceus* was more abundant during winter. The low number of stranding events in summer may be related to the weather conditions (i.e., the higher number of days with light or absence of wind and calm sea), since wind is an important factor influencing the stranding of marine fauna in the Strait of Messina [22]. The finding of mature females of *A. argo* with eggs laid in several seasons supports the hypothesis that this species is a “continuous spawner” [30,59,60]. Mature males examined in the present study were all found during the spring season. Furthermore, the finding of a mature female of the non-indigenous cephalopod *T. gracilis* gave us the opportunity to confirm that the eggs usually have an asynchronous development. In fact, the examination of the egg mass attached to the dorsal arm rod showed the presence of eggs and embryos at different stages, similar to what was observed by other authors [26,61]. The record of three females of *T. gracilis*, together with those reported so far in the Mediterranean [5], and in particular the evidence that the species breeds in this basin, suggest that the Mediterranean population of *T. gracilis* is now established, as hypothesized by Bello et al. [5].

The collection of numerous *A. argo* oothecae during the study period allowed us to examine a wide dimensional range of these structures (ShL ranged from 11.6 to 157 mm), although the maximum “shell” length for the species is estimated to be at least 300 mm [13]. Due to the large number of specimens, it was possible to examine the size relationships between different morphometric parameters of the *A. argo* ootheca for the first time, contributing to the current knowledge on this issue, which until now has been limited to Finn’s studies on *A. nodosus*, *A. hians* and *A. nouryi* [25,35]. Our data showed a good relationship between the morphometric parameters ApL-ShL, ShB-ShL and ApW-ShL, respectively, but the values of the coefficient of determination in the EW-ShL were lower. This result demonstrates the morphological variability of this portion (i.e., the distance between the extremities of the opposing “shell” ears, EW) of the ootheca. Several oothecae had fairly prominent ears, whereas in others this feature was barely noticeable, up to a total absence or even to a radical change in the curvature of this portion of the ootheca. In the 19th Century, these differences induced Monterosato [62] to consider this “variety” as a valid taxon, establishing the species *Argonauta cygnus,* now considered unaccepted. The variability of this portion of the ootheca was also observed by Finn [35] in other species of *Argonauta*, who deduced that ears may be formed or subsumed during the ootheca construction, resulting in transitional ootheca types. It is difficult to explain why these variations occur in ootheca ears, being so important to the physiology of the genus Argonauta. The ootheca is, after all, the protected place where all reproductive functions take place, from egg laying to fertilization of the eggs by the hectocotylus, to their hatching [14]. Indeed, Battaglia et al. [14], during their observation of an alive hectocotylus inside an empty stranded ootheca of *A. argo*, noticed that it was often clinging to the ootheca ears.

Morphometric analysis also allowed us to estimate the relationships between mantle length and beak size for the investigated species in Mediterranean waters, providing an updated useful tool for feeding ecology studies, where cephalopod prey items are often found partially or fully digested in stomach contents, and the only way to reconstruct prey size is to measure the lower beak and use these equations to assess cephalopod length. To date, similar relationships have been estimated for *A. argo* [29,37], *O. tuberculata* [29,33,36] and *T. violaceus* [29].

Our dataset also included Argonautoidea found in the stomach contents of large pelagic fish. These data indicate that holopelagic octopuses are more abundant than previously thought and, according to previous studies [15,17,18,63], large pelagic fish predators are reliable cephalopod collectors, which can provide scientists with important ecological and biological information about this taxon. It is known that these octopuses usually occur in epipelagic waters [24,64,65], although they can also reach the upper mesopelagic layer [13]. Therefore, the frequency of occurrence of Argonautoidea was higher in *T. belone* (%F= 28.8), which prefer to feed in epipelagic environments and we probably have a higher chance of encountering them than tuna or swordfish, which can also forage in deeper waters [63,66]. The finding of 330 holopelagic octopuses in a total of 800 stomachs of top predators, together with the records of stranded individuals, suggest that these species have important populations in the study area and that their supposed rarity should be reconsidered. According to Bello [67], in the last few decades, the “rare species status” of several Mediterranean cephalopods has been dismissed, due to an increase in sampling efforts at sea and in trophic ecology studies on teuthophagous predators, which both suggest that some species are not “rare”, but just “rarely caught”. This is particularly true for pelagic species, the sampling of which must be specially designed since, unlike other species, they rarely appear among the accidental catches of various fishing gear. Furthermore, conventional sampling gears used for monitoring the pelagic environment usually succeed in sampling juvenile individuals, whereas adult cephalopods generally avoid being caught [10]. With regard to Argonautoidea, the question of rarity remains unsolved for males. 

In addition to the previously stated reasons, the difficulty in collecting males may be due to several causes:

(i) A different life span between sexes, since it has been hypothesized that the male may die after detaching the hectocotylized arm and delivering it to the female [68,69,70,71]. Therefore, the male may survive for a short period (just enough time to mature and reproduce); this hypothesis is supported by the continuous reproductive period, which may allow for the turnover of population;
(ii) The female, after receiving the hectocotylus, could devour the male to obtain additional energy to complete egg maturation [14], thus reducing the possibility of sampling males;
(iii) The very small size of males makes them very elusive to sampling.

In this study, some Mediterranean individuals of holopelagic octopuses were genetically characterized using the mitochondrial fragment COI genetic marker, mostly used for the identification of cephalopod species [41,72,73,74]. 

Since the taxonomic identification of uncommon species by genetic markers is still poorly developed, a couple of primers dgLCO/dgHCO, have given the best results, obtaining good quality sequences. 

Most *A. argo* individuals investigated here using the barcoding approach best matched with the closest relative, *A. argo* LC596061 (GenBank repository), and the individual A006 showed higher similarity with *A. argo* AB191273 (GenBank repository). In both cases, the sequences of closest relatives seem to have been obtained by Japanese specimens, but unfortunately referred to an unpublished study; therefore, it is difficult to provide accurate evaluations. Recently, the NGS Illumina HiSeq approach was applied to gain more insight into the taxonomic position and lifecycle of *A. argo* found in Japanese waters, through full mitochondrial genome sequencing [75,76]. However, to the best of our knowledge, no data on full *A. argo* genomes are available for Mediterranean individuals.

Additonally, in the case of the *O. tuberculata* next relative, the location of the analysed individual is not specified. 

The correct identification of *Tremoctopus* spp. is a more problematic issue. It is known that several cases of misidentification between *T. violaceus* and *T. gracilis* have occurred in the past. The presence of *T. gracilis* in the Mediterranean basin was discovered by analysing a photo of an adult female in the Tyrrhenian Sea in August 2002, as discussed by Orsi Relini et al. [3,6]. After this event, a deeper analysis of previous records has shown that other individuals of *T. gracilis* had been erroneously ascribed to *T. violaceus* [3,5,6,41]. The study of Agus et al. [41] supported the hypothesis that errors/misidentifications are common in public repositories, probably because there are not many and easily applicable taxonomic characters to distinguish *T. violaceus* and *T. gracilis*. Other sources of variability in results may be due to the use of various genetic markers (e.g., COI, 16S, etc.) in different studies. The mitochondrial fragment 16S was also used as a DNA barcode marker for identifying *Tremoctopus* spp. [73]. In this last study, the authors noticed that individuals of the genus *Tremoctopus* had been confused in the past and inconsistently identified, attributing the causes of taxonomic uncertainty to the intrinsic limitations in obtaining specimens, the difficulties in distinguishing morphologically similar species and the limited number of genetic sequences currently available in GenBank for *T. violaceus* [73]. According to their results, *T. violaceus* only occurs in the Atlantic Ocean, whereas *T. gracilis* inhabits the Pacific and Indian oceans, suggesting that octopods from the Pacific were misidentified as *T. violaceus*, but were actually *T. gracilis*.

A substantial contribution concerning the *Tremoctopus* species in the Mediterranean Sea was provided by Agus et al. [41], reporting morphologic and genetic characterization of Mediterranean sequences clustering with the sequences of *Tremoctopus violaceus* from the Gulf of Mexico. These authors analysed the sequences of two mtDNA genes (COI and 16S), strongly recommended the integration of a genetic approach for the description of these octopods and confirmed the occurrence of *T. gracilis* in the Mediterranean area. These findings are in line with the results reported in our study. The complete mitochondrial genome and the phylogenetic position of the pelagic octopus *Tremoctopus violaceus* from Taiwan has also been provided [77], but Jiménez-Badillo et al. [73] and Agus et al. [41] concluded that this specimen should be attributed to *T. gracilis*. Indeed, these studies [41,73] proposed that several individuals classified as *T. violaceus* were actually *T. gracilis* specimens. Despite this, these sequences were still deposited as *T. violaceus* on GenBank repositories. All these aspects, in addition to the few taxonomic differences between species and the difficulty in finding intact individuals to improve taxonomic analysis, make it extremely difficult to conduct the taxonomic identification of some rare individuals by genetic analysis alone, and a lot of studies are still necessary in the future. In our study, all specimens of *Tremoctopus* sp. were strictly related to the sequences deposited by Agus et al. [41], obtained from Mediterranean individuals. The only exception was represented by the individual TV011, which matched better with sequences of individuals from the Gulf of Mexico. This could suggest genetic heterogeneity and variability within *Tremoctopus* sp. populations in the Mediterranean Sea. 

TV008, i.e., one of the two small immature males of *Tremoctopus*, resulted in being strictly related to *T. violaceus*; therefore, the genetic results suggest that these two males found on the same date and in the same location may be ascribed to the species *T. violaceus*.

The sequences obtained from the two *T. gracilis* specimens (TG002, TG003) were 99% similar to each other, supporting morphological identification, which clearly established that the two individuals were *T. gracilis*. The sequences obtained in this study from the two *T. gracilis* individuals did not show similarities with the *T. gracilis* individuals described in the study of Agus et al. [41], isolated from different study areas. Differently, they showed high similarity with the two Genbank deposited sequences, MW351786.1 and MW351787.1, not included in the analysis by Agus et al. [41], obtained from individuals from the Gulf of Mexico. However, MW351786.1 and MW351787.1 have not been reported in a published paper, so it is not possible to ascertain the correct identification of those individuals.

The highly intra-specific variability of the fragment COI is an aspect that could make a proper phylogenetic analysis difficult, even in consideration of the small number of deposited sequences and the potential genetic divergences due to geographical factors. The fragmentation of the available information in relation to the deposited sequences is a further critical factor for those who study these species. Further studies are planned to use at least an additional genetic marker in addition to COI to obtain more reliable results. 

## 5. Conclusions

The present paper represents a great effort in the study of Mediterranean holopelagic octopods belonging to Argonautoidea. The importance of collecting information from different sources of data is strictly related to the use of a multidisciplinary approach, which improves knowledge on several aspects of the ecology, biology and distribution of these poorly known species.

The results reported here suggest that the use of an integrated approach based on morphological and molecular identification, in a mutually supportive key, should be considered of paramount importance. In our opinion, and as also shown by our results, genetic study alone is still inconsistent in achieving a correct identification of a species, but it is, however, important to add information and data in this scantly investigated field. In conclusion, the morphological and genetic information provided in this study provides an important contribution to further evolutionary analysis of rare cephalopod species. Further efforts should be devoted to the collection of new specimens, in particular where different species of congeneric pelagic octopuses coexist, with the aim of deepening taxonomical and genetics features to better clarify the distribution of species populations and provide clearer tools for species identification. The misidentifications of *Tremoctopus* spp. in the past, the contrasting data in terms of morphological features and genetics outputs also suggest a last intriguing hypothesis that cannot be excluded a priori; i.e., the potential existence of cases of interspecific hybrids between closer species.

## Figures and Tables

**Table 1:** Sample IDs, accession number and species identification assessed using the primer set Fish F2/Fish R2.

Sample	AN	Taxonomy	Sim	Taxonomy	Sim
		BLASTn Search Implemented in GenBank	(%)	BOLD-IDS	(%)
				Search	
AA005	-	nd	nd	nd	nd
AA015	-	nd	nd	nd	nd
AA015E	-	nd	nd	nd	nd
AA006	-	nd	nd	nd	nd
AA006E	-	nd	nd	nd	nd
AA006U	-	nd	nd	nd	nd
AA007	-	nd	nd	nd	nd
AA008	-	nd	nd	nd	nd
OT005	-	nd	nd	nd	nd
OT005E	OP132818	AY557519, *Ocythoe tuberculata*	100	*Ocythoe tuberculata*, KY947106	100
TG002	OP132821	OM025233, *Tremoctopus violaceus* isolate TVBA	98	*Tremoctopus*	97
				*violaceus*, private sequence	
TG002U	-	nd	nd	nd	nd
TG003	-	nd	nd	nd	nd
TV006	OP132819	OM025233, *Tremoctopus violaceus* isolate TVBA	98	*Tremoctopus*	85
				*violaceus*, private sequence	
TV008	-	nd	nd	nd	nd
TV011	OP132820	OM025233, *Tremoctopus violaceus* isolate TVBA	99	*Tremoctopus*	99
				*violaceus*, private sequence	

AN, Accession Number; Sim, Similarity; nd, not determined.

**Table 2:** Sample IDs, accession number and species identification assessed using the primer set dgLCO/dgHCO.

Sample	AN	Taxonomy	Sim	Taxonomy	Sim
		BLASTn Search Implemented in GenBank	(%)	BOLD-IDS	(%)
				Search	
AA005	OP132805	LC596061, *Argonauta argo* UMUT:RM33391	100	BOLD:AAJ8256, *Argonauta argo*	99.8
AA015	OP132806	LC596061, *Argonauta argo* UMUT:RM33391	100	*Argonauta*	100
				*hians*, private sequence	
AA015E	-	nd	nd	nd	nd
AA006	OP132807	AB191273, *Argonauta argo* mitochondrial COI gene	100	BOLD:AAJ8256, *Argonauta argo*	89
AA006E	OP132808	LC596061, *Argonauta argo* UMUT:RM33391	100	*Argonauta*	100
				*hians,* private sequence	
AA006U	-	nd			
AA007	OP132809	LC596061, *Argonauta argo* UMUT:RM33391	100	BOLD:AAJ8256, *Argonauta argo*	99.8
AA008	OP132810	LC596061, *Argonauta argo* UMUT:RM33391	100	*Argonauta*	99.8
				*hians,* private sequence	
OT005	OP132811	KY947106, *Ocythoe tuberculata* isolate ESFRI-octo60	99	nd	nd
OT005E	OP132812	AY557519, *Ocythoe tuberculata* cytochrome c oxidase subunit I gene	100	BOLD:ADR2058, *Ocythoe tuberculata*	100
TG002	OP132816	MW351787, *Tremoctopus violaceus* isolate *T. violaceus* 2	99	*Tremoctopus violaceus,* private sequence	100
TG002U	-	nd	nd	nd	nd
TG003	OP132817	MW351787, *Tremoctopus violaceus* isolate *T. violaceus* 2	100	nd	nd
TV006	OP132813	MW351787, *Tremoctopus violaceus* isolate T. violaceus 2	98	nd	nd
TV008	OP132814	OM025233, *Tremoctopus violaceus* isolate TVBA	100	*Tremoctopus violaceus,* private sequence	97
TV011	OP132815	MW351787, *Tremoctopus violaceus* isolate *T. violaceus* 2	99	nd	nd

AN, Accession Number; Sim, Similarity; nd, not determined.

## Data Availability

The data presented in this study are available on request from the corresponding author.

## Supplementary Materials

The following supporting information can be downloaded at: https://www.mdpi.com/article/10.3390/biology12030420/s1, Table S1: List of fresh Argonautoidea specimens collected; Table S2: Total number of predator stomachs used in this study to collect data on the ingestion of holopelagic octopuses (ALB = albacore; BFT = bluefin tuna; MSP = Mediterranean spearfish; SWO = swordfish); Table S3: List of the individuals considered for genetic analysis; Figure S1: Relationship between beak size (lower hood length LHL) and mantle length (ML) in Argonauta argo (A), Ocythoe tuberculata (B) and Tremoctopus violaceus (C); Figure S2: Relationship between “shell” lenght (ShL) and dorsal mantle length for Argonauta argo; Figure S3: Number of cephalopod Argonautoidea found in the stomachs of pelagic predators (ALB = albacore; BFT = bluefin tuna; MSP = Mediterranean spearfish; SWO = swordfish); Table S4: Details of the COI sequences used in this study to compute phylogenetic tree of *Tremoctopus* spp. individuals. Figure S4: Number of cephalopod Argonautoidea found in the stomachs of pelagic predators (ALB = albacore; BFT = bluefin tuna; MSP = Mediterranean spearfish; SWO = swordfish). References [78,79,80,81,82,83] are cited in the supplementary materials or in the main text.
