# Supplementary material for: How Rare Are Argonautoidea Octopuses in the Mediterranean? New Data from Stranding Events, Stomach Contents and Genetics
Source: Biology (Basel). 2023 Mar 9;12(3):420. doi: 10.3390/biology12030420 (PMC10045602; doi:10.3390/biology12030420)
Supplement: Supplementary file 1 [file biology-12-00420-s001.zip › biology-2176029-supplementary.pdf]

biology

**SUPPLEMENTARY MATERIALS**

**Battaglia et al. - How rare are Argonautoidea octopuses in the Mediterranean? New data from stranding events, stomach contents and genetics.**

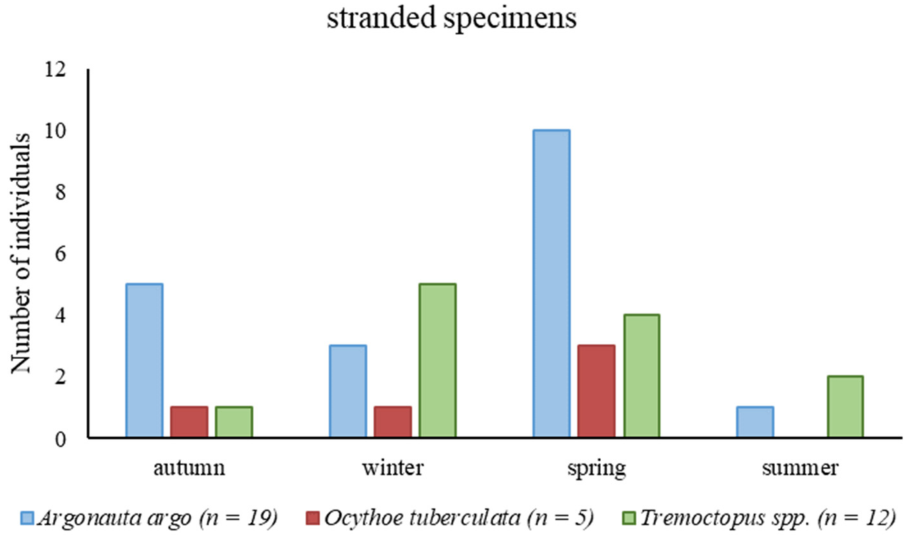


Figure S1. Number of stranded specimens of *Argonauta argo*, *Ocythoe tuberculata* and *Tremoctopus violaceus* for each season.

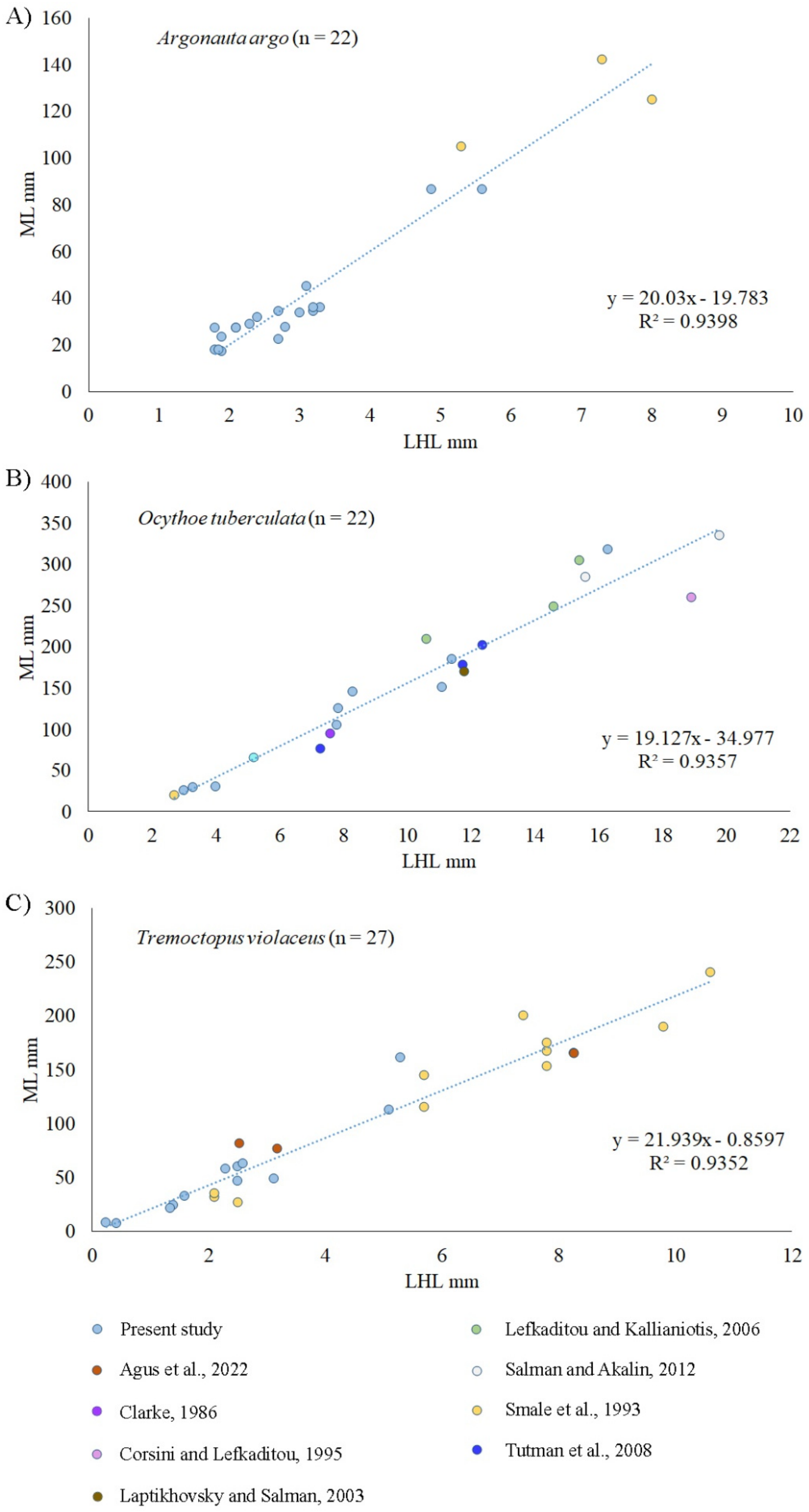


Figure S2. Relationship between beak size (lower hood length LHL) and mantle length (ML) in *Argonauta argo* (A), *Ocythoe tuberculata* (B) and *Tremoctopus violaceus* (C).

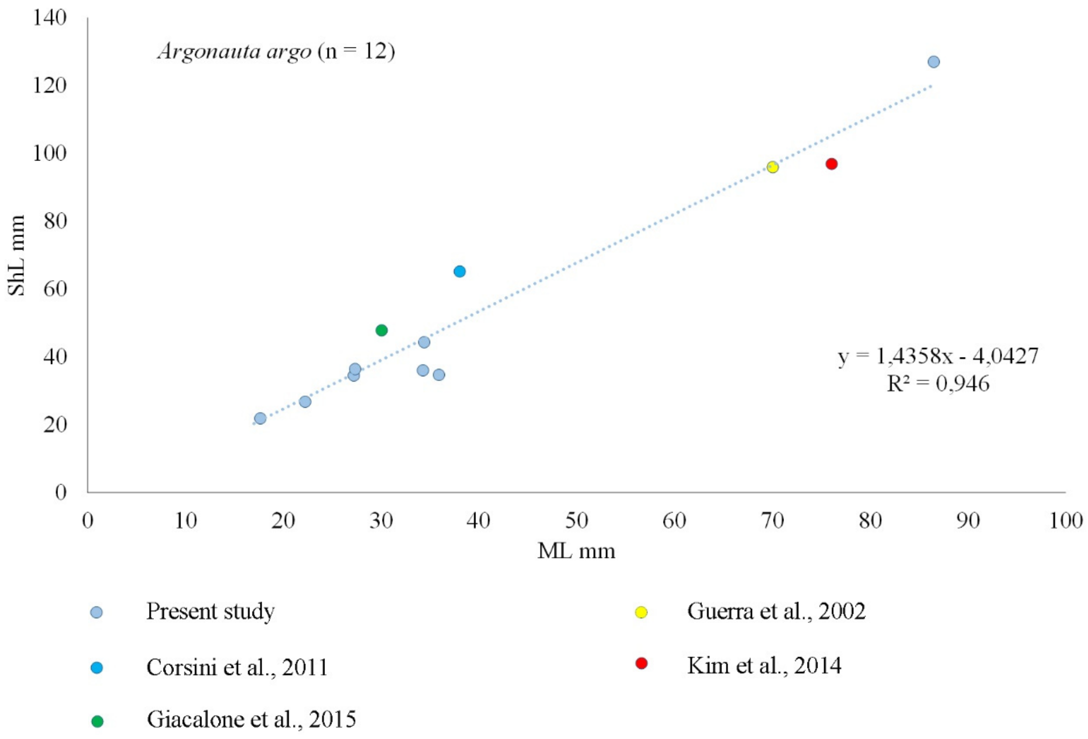


Figure S3. Relationship between shell lenght (ShL) and dorsal mantle length for *Argonauta argo*.

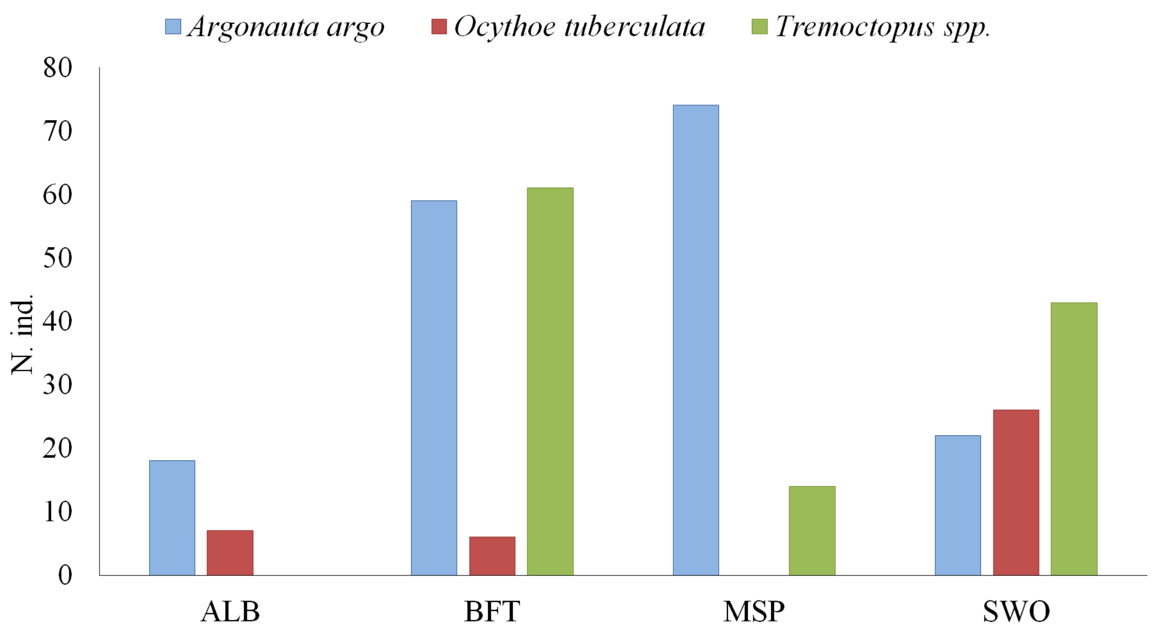


Figure S4. Number of cephalopod Argonautoidea found in the stomachs of pelagic predators (ALB = albacore; BFT = bluefin tuna; MSP = Mediterranean spearfish; SWO = swordfish)

Table S1. List of fresh Argonautoidea specimens collected.

| Species | Code | Area | Date | ML mm | Sex |
|---|---|---|---|---|---|
| *Argonauta argo* | AA 001 | Strait of Messina | 24/03/2009 | 31.6 | F |
| | AA 002 | Strait of Messina | 16/11/2008 | 23.2 | F |
| | AA 003 | Strait of Messina | 19/05/2010 | 27.2 | F |
| | AA 004 | Strait of Messina | 19/05/2010 | 27.3 | F |
| | AA 005 | Strait of Messina | 05/03/2011 | 86.4 | F |
| | AA 006 | Strait of Messina | 28/10/2011 | 86.5 | F |
| | AA 007 | Strait of Messina | 10/11/2012 | 27.6 | F |
| | AA 008 | Strait of Messina | 03/04/2012 | 45.1 | F |
| | AA 009 | Strait of Messina | 06/07/2012 | 34.4 | F |
| | AA 010 | Strait of Messina | 20/06/2019 | 17 | F |
| | AA 011 | Strait of Messina | 06/05/2020 | 17.8 | F |
| | AA 012 | Strait of Messina | 19/01/2014 | 27.3 | F |
| | AA 013 | Strait of Messina | 21/10/2020 | 17.65 | F |
| | AA 014 | Strait of Messina | 11/04/2021 | 22.2 | F |
| | AA 015 | Strait of Messina | 07/02/2021 | 34.4 | F |
| | AA 016 | Strait of Messina | 06/04/2018 | 35.9 | F |
| | AA 018 | Strait of Messina | 08/05/2016 | 35.9 | F |
| | AA 019 | Strait of Messina | 08/05/2016 | 33.6 | F |
| | AA 020 | Strait of Messina | 15/11/2016 | 28.7 | F |
| | AA 021 | Strait of Messina | spring 1982 | 6.161 | M |
| *Ocythoe tuberculata* | OT 001 | Milazzo | 17/03/2009 | 145.6 | F |
| | OT 002 | Strait of Messina | 11/05/2008 | 24.1 | M |
| | OT 003 | Gulf of Patti | spring 2017 | 150.42 | F |
| | OT 004 | Finale di Pollina | 29/03/2021 | 318 | F |
| | OT 005 | Milazzo | 17/02/2018 | 125.1 | F |
| | OT 006 | Strait of Messina | 11/05/2008 | 28.9 | F |
| | OT 007 | Strait of Messina | 17/06/1996 | 25 | F |
| | OT 008 | Strait of Messina | 13/03/2002 | 30 | F |
| | OT 009 | Strait of Messina | 30/09/2012 | 105 | F |
| *Tremoctopus violaceus* | TV 001 | Strait of Messina | winter 1980 | 46.4 | F |
| | TV 002 | Strait of Messina | winter 1980 | 48.5 | F |
| | TV 003 | Strait of Messina | 23/08/2020 | 112.8 | F |
| | TV 004 | Strait of Messina | 20/01/2021 | 7 | F |
| | TV 005 | Strait of Messina | 21/01/2021 | 8 | F |
| | TV 006 | Strait of Messina | 22/01/2021 | 32.75 | F |
| | TV 009 | Strait of Messina | 03/07/2015 | 23.8 | F |
| | TV 010 | Strait of Messina | 05/07/2012 | 21.3 | F |
| | TV 011 | Strait of Messina | 19/12/2021 | 161 | F |
| | TV 012 | Strait of Messina | 20/06/1999 | 60 | F |
| | TV 013 | Strait of Messina | 20/06/1999 | 63 | F |
| | TV 014 | Strait of Messina | 20/06/1999 | 58 | F |
| | TV 015 | Strait of Messina | 15/05/2005 | 26.1 | M |
| *Tremoctopus gracilis* | TG 001 | Milazzo | 17/01/2005 | 166 | F |
| | TG 002 | Strait of Messina | 18/08/2014 | 150 | F |
| | TG 003 | Mongiove | 12/02/2022 | 70.3 | F |
| *Tremoctopus* sp. | TV 007 | Strait of Messina | 23/02/2021 | 7.5 | M |
| | TV 008 | Strait of Messina | 23/02/2021 | 7.9 | M |

Table S2. Total number of predator stomachs used in this study to collect data on the ingestion of holopelagic octopuses (ALB = albacore; BFT = bluefin tuna; MSP = Mediterranean spearfish; SWO = swordfish).

| Predator | Years | n. stomachs | Size range (cm) | Size type | Reference study |
|---|---|---|---|---|---|
| ALB | 2005-2006 | 127 | 38.9 - 102.1 | FL | [18] and unpublished data |
| BFT | 1998-2000 | 139 | 6.3 - 49.5 | TL | [16] |
| BFT | 2010-2011 | 123 | 115 - 222 | FL | [63] |
| BFT | 2011 | 5 | 110 - 145 | FL | Unpublished data |
| BFT | 2021 | 34 | 96 - 207 | FL | Unpublished data |
| MSP | 2020 | 58 | 113 - 196 | LJFL | [78] |
| MSP | 2004-2006 | 53 | 105 - 195 | LJFL | [66] |
| SWO | 2004-2006 | 103 | 110 - 210 | LJFL | [66] |
| SWO | 2008 | 68 | 72 - 216 | LJFL | Unpublished data |
| SWO | 2019 | 65 | 114 - 206 | LJFL | Unpublished data |
| SWO | 2021 | 25 | 111 - 184 | LJFL | Unpublished data |

Tab. S3. List of the individuals considered for genetic analysis.

| Sample Code | Sampling area | Species | Tissue type |
|---|---|---|---|
| AA005 | Strait of Messina | *Argonauta argo* | Muscle |
| AA015E | Strait of Messina | *Argonauta argo* | Muscle (hectocotylus inside shell) |
| AA006 | Strait of Messina | *Argonauta argo* | Muscle |
| AA006E | Strait of Messina | *Argonauta argo* | Muscle (hectocotylus inside shell) |
| AA006U | Strait of Messina | *Argonauta argo* | Eggs |
| AA007 | Strait of Messina | *Argonauta argo* | Muscle |
| AA008 | Strait of Messina | *Argonauta argo* | Muscle |
| AA015 | Strait of Messina | *Argonauta argo* | Muscle |
| OT005 | Milazzo | *Ocythoe tuberculata* | Muscle |
| OT005E | Milazzo | *Ocythoe tuberculata* | Muscle (hectocotylus inside mantle) |
| TG002 | Strait of Messina | *Tremoctopus gracilis* | Muscle |
| TG002U | Strait of Messina | *Tremoctopus gracilis* | Eggs with embryos |
| TG003 | Strait of Messina | *Tremoctopus gracilis* | Muscle |
| TV006 | Strait of Messina | *Tremoctopus violaceus* | Muscle |
| TV008 | Strait of Messina | *Tremoctopus sp.* | Muscle |
| TV011 | Strait of Messina | *Tremoctopus violaceus* | Muscle |

Table S4. Details of the COI sequences used in this study to compute phylogenetic tree of cephalopod individuals.

| GenBank AN | Taxonomy in GenBank | Sampling area | Reference |
|---|---|---|---|
| MW351786 | *Tremoctopus violaceus* | Yucatan peninsula, southern Gulf of Mexico | Santana-Cisneros, unpublished |
| MW351787 | *Tremoctopus violaceus* | Yucatan peninsula, southern Gulf of Mexico | Santana-Cisneros, unpublished |
| OM025233 | *Tremoctopus violaceus* | Apulia, Italy —Mediterranean Sea | [41] |
| MN443917 | *Tremoctopus violaceus* | South Korea—Pacific Ocean | Kim, unpublished |
| MN370032 | *Tremoctopus gracilis* | - | Sajikumar et al., unpublished |
| MW025168 | *Tremoctopus violaceus* | South Korea—Pacific Ocean | Oh et al., unpublished |
| MH379648 | *Tremoctopus gracilis* | India—Indian Ocean | Geetha et al., unpublished |
| MN370034 | *Tremoctopus gracilis* | - | Sajikumar et al., unpublished |
| MN370033 | *Tremoctopus gracilis* | - | Sajikumar et al., unpublished |
| KY649286 | *Tremoctopus violaceus* | Taiwan—Pacific Ocean | [71] |
| AF377978 | *Tremoctopus violaceus* | Florida, U.S.A.—Atlantic Ocean | [78] |
| MN560197 | *Tremoctopus* sp. | Chile—Pacific Ocean | [79] |
| MK186001 | *Tremoctopus robsoni* | New Zealand – Pacific Ocean | [80] |
| AY557519 | *Ocythoe tuberculata* | - | [81] |
| KY947106 | *Ocythoe tuberculata* | Korea | [82] |
| LC596061 | *Argonauta argo* | Oki Island, Japan | [75] |

## Reference


78. AA.VV. *Valutazione dello stato e della distribuzione dell'aguglia imperiale (Tetrapturus belone)*; Relazione finale, Misura a titolarità 1.40 lettere c) e i) del PO FEAMP 2014/2020; 2021. Available online: https://www.regione.sicilia.it/sites/default/files/2022-03/Elenco%20misura%201.40%20al%2031.12.2021%20-%20anno%202021.pdf (accessed on 15 october 2022).
79. Carlini, D.B.; Young, R.E.; Vecchione, M. A molecular phylogeny of the Octopoda (Mollusca: Cephalopoda) evaluated in light of morphological evidence. *Mol. Phylogenet. Evol.* **2001**, *21*, 388–397. https://doi.org/10.1006/mpev.2001.1022.
80. Carrasco, S.A.; Varela, A.I.; Ibáñez, C.M.; Sellanes, J.; Thiel, M. Paralarval and juvenile stages as a proxy for cephalopod diversity in the Juan Fernández and Desventuradas ecoregion, southeast Paific Ocean. *Bull. Mar. Sci.* **2020**, *96*, 263–280. https://doi.org/10.5343/bms.2019.0055.
81. Braid, H.E.; Bolstad, K.S. Cephalopod biodiversity of the Kermadec Islands: Implications for conservation and some future taxonomic priorities. *Invertebr. Syst.* **2019**, *33*, 402–425.
82. Lindgren, A.R.; Giribet, G.; Nishiguchi, M.K. A combined approach to the phylogeny of Cephalopoda (Mollusca). *Cladistics* **2004**, *20*, 454–486. https://doi.org/10.1111/j.1096-0031.2004.00032.x.
83. Kim, J.B.; Yang, J.H.; Kim, Y.; Lee, S.J. First record of a rare pelagic octopod, *Ocythoe tuberculata* (Cephalopoda: Ocythoidae), in Korea. *Anim. Cells Syst.* **2017**, *21*, 419–423. https://doi.org/10.1080/19768354.2017.1377766.
